# A welder case with secondary haemochromatosis, which rarely accompanies pneumoconiosis

**DOI:** 10.1093/occmed/kqaf030

**Published:** 2025-06-10

**Authors:** S Çakmakcı Karakaya, Y S Hasanlı, A U Demir

**Affiliations:** Subdivision of Work and Occupational Diseases, Department of Public Health, Hacettepe University Faculty of Medicine, Ankara, Türkiye; Subdivision of Work and Occupational Diseases, Department of Internal Diseases, Ministry of Health Etlik City Hospital, Ankara, Türkiye; Department of Pulmonary Diseases, Hacettepe University Faculty of Medicine, Ankara, Türkiye

## Abstract

Excessive iron accumulation in the body can cause serious health problems, yet occupational factors have not been extensively addressed in the research literature. Welding fumes contain iron, which can be absorbed by inhalation, possibly leading toiron overload and impaired iron homeostasis in welders. We present a case of a welder with secondary haemochromatosis and welder’s lung after more than 20 years in the metal industry. This highlights the need for risk management and comprehensive health monitoring, including ferritin and transferrin saturation, in pre-employment health assessment and periodic workplace health surveillance. Regular monitoring, early detection and proper workplace safety protocols can reduce the risk. Addressing both pulmonary and systemic risks through multidisciplinary evaluations is essential. We aimed to provide a new perspective by emphasizing iron homeostasis assessment in welders, which may help prevent underdiagnosed cases of occupational iron overload and promote targeted preventive strategies.

## Background

Iron (Fe) is essential for human metabolism and is absorbed through the gastrointestinal (GI) tract and eliminated via faeces, while the lungs retain it in a less reactive form for detoxification [1]. Excess iron binds to ferritin and is stored in the liver to prevent oxidative damage. Serum ferritin reflects total iron stores especially in the absence of inflammation or cancer [2, 3]. However, iron overload can be toxic.

Primary haemochromatosis involves excessive iron absorption from enterocytes, while secondary haemochromatosis stems from chronic erythropoiesis disorders, porphyrin biosynthesis defects, hepatitis C or prolonged high iron intake [4]. Welding fumes, primarily ferric oxide (Fe_2_O_3_) [5], raise concerns about welders developing iron overload and disrupted homeostasis via the Fenton reaction [6]. Welder’s lung and pulmonary siderosis are linked to prolonged exposure, with elevated ferritin in serum and bronchoalveolar lavage (BAL) [7] and hemosiderin-laden alveolar macrophages (HLMs) observed in BAL and lung biopsies, suggesting systemic iron accumulation through both inhalation and GI absorption [8]. Literature on occupational iron overload in welders is limited and lacks haemochromatosis gene testing. We wanted to contribute to the literature with this case.

## Case presentation

A 48-year-old male, diagnosed with hypersensitivity pneumonitis (HP) was referred for worsening diffuse centrilobular ground glass opacities on thorax computed tomography (CT). He worked as a gas welder in the metal industry in Türkiye from 1999 to 2023, making metal railings using iron raw materials and occasionally cutting with a spiral stone, without additional chemical processing, wiping, painting, cleaning or sanding. Although he wore appropriate protective gear, he lacked respiratory protection. The workplace had inadequate ventilation and the process created dense smoke and dust.

He quit smoking 12 years previously after a 20-pack/year history, did not use alcohol or drugs, had exposure to biomass until 10 years ago and white soil molasses production in early childhood. He was diagnosed with HP in 2016 due to 1-year budgerigar exposure and subsequently ceased contact with the bird. His medical history included hyperlipidaemia and fatty liver, but no regular medication use.

He was asymptomatic with a normal vital sign including oxygen saturation of 96% at 930-m altitude. His physical examination was unremarkable, except for a smooth-edged, palpable liver.

## Investigations

Lab results showed elevated alanine aminotransferase (ALT) (208 U/L), gamma-glutamyl transferase (214 U/L), normal aspartate aminotransferase (46 U/L), alkaline phosphatase (89 U/L) and total bilirubin (0.53 mg/dl). Other values included erythrocyte sedimentation rate (5 mm/h), red blood count (5.48 × 10^6^/µl), white blood cell (7.83 × 10^3^/µl), platelets (183 × 10^3^/µl), and haemoglobin (16.2 g/dl), haematocrit (50.4%), iron (129 µg/dl;N:50-150), total iron binding capacity (313 µg/dl;N:228-428), transferrin saturation (TS; 41%;N:20-50) and ferritin (819 µg/l;N:20-336). Urinalysis was normal. The blood heavy metal (arsenic, mercury, cadmium, cobalt, lead, manganese) was within normal limits. Pulmonary function test indicated minor airway obstruction, though diffusing capacity for carbon monoxide could not be measured.

**Figure 1.**
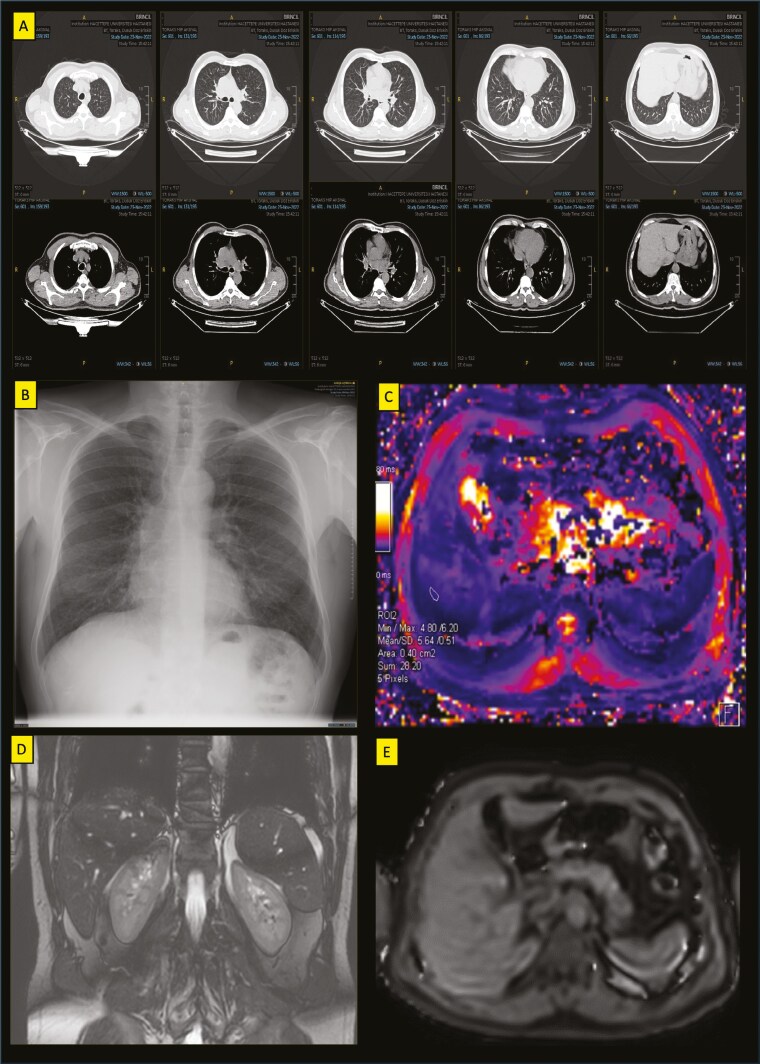
Various sections of thorax CT findings (A), Posteroanterior chest radiography (B), Thalassemia iron accumulation MRI (Liver Section) Liver T2* value on the T2 star map is approximately 5.6 ms and decreased in accordance with minimal iron accumulation (C), abdomen dynamic MRI, T2 liver and spleen are clearly black (hypointense) (D) T2* sequence, liver parenchyma T2* measurement value is 4.9 ms, compatible with significant iron accumulation (E).

Thorax CT revealed progression of diffuse centrilobular opacities, subsegmental atelectasis and bronchial enlargement (Figure 1A). Chest X-ray was consistent with pneumoconiosis (s/s 1/0) (Figure 1B). Beta-thalassemia quantitative magnetic resonance imaging (MRI) revealed no myocardial iron accumulation (T2*24 ms) but minimal liver iron accumulation (T2*5.6 ms) (Figure 1C). Thereupon, dynamic abdominal MRI showed mild hepatomegaly (right lobe 17 cm), with normal liver function, no focal lesions and advanced liver (T2*4.9 ms) and spleen iron accumulation (Figure 1D-E).

**Figure 2.**
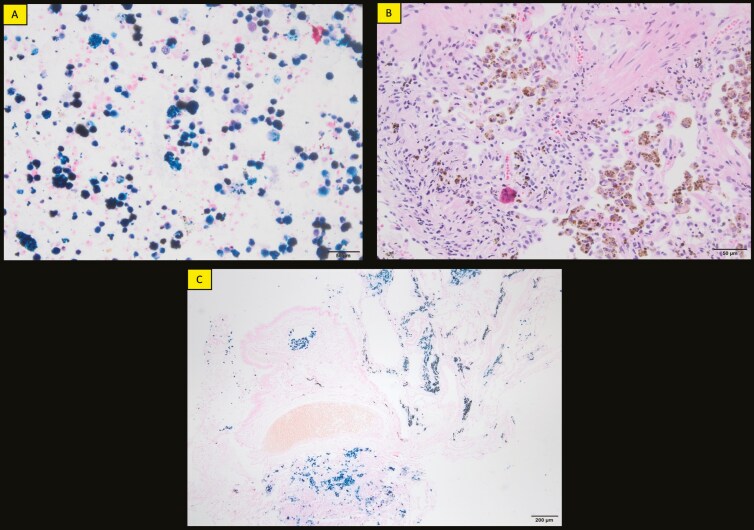
BAL Cytology: Significant intracytoplasmic hemosiderin accumulation is observed in nearly all macrophages (Prussian blue staining, ×200) (A). Forceps biopsy pathology: Biopsy from the lingula/middle lobe obtained via FOB shows many intraalveolar macrophages laden with hemosiderin pigment along with mild, non-specific fibroinflammatory changes (H&E staining, brown, ×200) (B). Hemosiderin pigment changes (Prussian blue staining, ×40) (C).

Fiberoptic bronchoscopy (FOB) revealed dense hemosiderin accumulation in nearly all macrophages with Prussian blue (Figure 2A), rare polymorphous nucleated leukocytes in BAL cytology. Cultures and tuberculosis panel, including RT-PCR, were negative. CD4/CD8 ratio was unavailable. BAL cytology showed 80% macrophages, 15% neutrophils and leukocytes and 5% lymphocytes. Oil Red O staining showed no significant lipid accumulation. Biopsies from the lingula/right middle lobe displayed HLMs and mild non-specific fibroinflammatory changes, with no granulomatous inflammation or neoplasia observed (Figure 2B and C).

As the bird exposure ended in 2016, HP was unlikely. Based on findings, pneumoconiosis due to welding was diagnosed. Due to MRI findings, HFE gene mutation analysis (exons 2, 3, 4 and exon-intron compounds) was conducted for differential diagnosis of primary/secondary haemochromatosis, and no mutation was detected. Consequently, secondary haemochromatosis accompanying pneumoconiosis was considered, likely due to cumulative iron inhalation from years of welding. Occupational health and safety recommendations were made, along with legal notification.

## Discussion

This case highlights a welder developing systemic iron overload after years of poor occupational safety, resulting in welder’s lung. Systemic iron overload was detected with evaluations revealed occupational exposure as the cause. Iron absorption was confirmed through lung macrophages and BAL, showing HLMs, a common finding in such cases, with lung biopsy supporting the diagnosis by showing abundant HLMs in the interstitial peribronchovascular areas [8–10]. These tests also helped rule out differential diagnoses.

Excessive iron accumulation, as seen in primary haemochromatosis, can cause arthropathy, diabetes, cardiomyopathy, gonadal dysfunction, neuroferritinopathy, Parkinson’s, Alzheimer’s, chronic liver disease and hepatocellular carcinoma (HCC) and serious symptoms such as weakness and lethargy, chronic and widespread pain and skin hyperpigmentation [4]. These conditions severely impact workers’ health and quality of life, diminishing earning potential and causing long-term health problems post-retirement.

Depending on the welding technique, welders may be exposed to high levels of respirable iron. The WELDOX study involving 192 welders showed that respirable iron levels above 1800 µg/m³ iron homeostasis, leading to elevated serum ferritin and prohepcidin levels [2]. Monitoring serum ferritin may help identify iron overload in welders, and using respiratory protection (particularly purified air supply) can significantly reduce exposure.

A recent study of 20 welders supports an occupational form of hepatic iron overload linked to long-term welding fume exposure [11]. Liver MRI or biopsy confirmed iron overload with no significant correlation to age, exposure duration or smoking. Serum ferritin exceeded 1000 ng/ml in 90% of welders, and TS levels were above 45% in 60%. Significant correlations emerged between serum ferritin and liver MRI findings, and ALT levels. Liver steatosis was observed in 65% of cases.

Studies on hereditary haemochromatosis mutations have not conclusively ruled out a link between systemic iron overload and haemochromatosis. In the latter study, three patients were heterozygous for the p.His63Asp variant and one for the p.Cys282Tyr in HFE, with no mutations in other haemochromatosis-related genes (TFR2, SLC40A1, HAMP, HFE2) [11]. Three reported cases of welder’s siderosis suggested that systemic iron excess could result from occupational exposure, undiagnosed hereditary haemochromatosis or both [12]. The findings imply that siderosis may cause systemic iron overload, supported by correlations between hepatic enzymes and serum ferritin [13]. While no homozygous HFE mutations were identified, one high-exposure welder with elevated iron markers showed heterozygous mutations at both loci, suggesting that HFE mutations may elevate ferritin independently or with occupational exposure [2].

In 1996, Feder *et al.* [14] identified a homozygous mutation in an MHC class I-like gene in most hereditary haemochromatosis cases. Since then, genotyping for HFE mutations, particularly C282Y, has become a standard for suspected hereditary haemochromatosis or elevated iron levels. H63D and S65C are also common HFE mutations, and clinical labs typically screen for all three [15]. The H63D mutation, more common in Asian populations, is considered a primary cause of hereditary haemochromatosis in Türkiye [16, 17]. However, these mutations were absent in our patient.

International Labour Organization and World Health Organization have set occupational exposure limits for Fe_2_O_3_, with TLV of 5 mg/m^3^ for respirable iron [18]. The value specified in the national legislation annex is also the same [19]. Measuring airborne iron oxide is crucial for risk management, yet no data were available from the patient’s workplace. His prolonged exposure to iron fumes, poor ventilation and lack of respiratory protection likely contributed to his condition, which is also a violation of the current national legislation regarding protective equipment [19].

Although there is no direct evidence linking airborne iron oxides to lung cancer, iron and steel foundries are classified as carcinogenic to humans by the International Agency for Research on Cancer [20]. This underscores the importance of cancer risk evaluation (HCC, lung and colon cancer) and regular health checks for workers, even post-retirement. We recommended a 6-month follow-up at a pulmonary and occupational clinic post-workplace change to monitor clinical findings, advised against tobacco use and planned haematology follow-up for secondary haemochromatosis.

Few sources address both clinical and occupational health aspects, though prolonged welding fume exposure is proposed as a rare, acquired cause of hyperferritinemia and liver iron overload. Literature suggests the need for follow-up post-retirement, as many welders wish to continue working. Examining welders with and without siderosis and studying iron fume exposure correlations with serum iron markers could clarify the impact on total body iron stores. Research is needed in this context. But, most importantly, occupational health and safety controls should prioritize risk elimination.

Patient history, clinical evaluation, lab tests, genetic analysis, FOB and radiology confirmed secondary haemochromatosis, marking Türkiye’s first official case from occupational exposure. Long-term welding fume exposure should be considered an acquired cause of liver iron overload. Regular monitoring of iron markers in welders during health checks will help identify those at risk and apply necessary protective measures. Given the multi-organ impact, a multidisciplinary approach is essential for those unaware of the occupational risks.

**Key learning points**

*What is already known about this subject:*

- Lung pathologies may develop in people who have been exposed to welder smoke for many years.
- Although the causes of secondary haemochromatosis are well defined, case reports regarding exposure to welding fumes, which should be among the causes, have begun to be published and this presentation supports this.

*What this study adds:*

- A person with welder’s lung may have systemic iron exposure, and this should be a warning for workplace physicians to refer them to a hospital for multidisciplinary evaluation.
- Routinely checking blood ferritin, transferrin saturation during periodic examinations of welders can help detect and prevent diseases before they reach systemic involvement.

*What impact this may have on practice or policy:*

- In people with or without symptoms, being diagnosed with other diseases and being followed for many years and missing occupational diseases prevent control at the source and cause the person to continue working in the same environment.
- A detailed occupational history and questioning of work environments can be lifesaving.
